# Comparison of DeNovix, NanoDrop and Qubit for DNA quantification and impurity detection of bacterial DNA extracts

**DOI:** 10.1371/journal.pone.0305650

**Published:** 2024-06-17

**Authors:** Nick Versmessen, Leen Van Simaey, Abel Abera Negash, Marjolein Vandekerckhove, Paco Hulpiau, Mario Vaneechoutte, Piet Cools

**Affiliations:** 1 Laboratory Bacteriology Research, Department of Diagnostic Sciences, Faculty of Medicine and Health Sciences, Ghent University, Ghent, Belgium; 2 Armauer Hansen Research Institute, Addis Ababa, Ethiopia; 3 Department of Microbiology, Immunology and Parasitology, Addis Ababa University, Addis Ababa, Ethiopia; 4 HOWEST University of Applied Sciences, Bruges, Belgium; Universidade Lisboa, Instituto superior Técnico, PORTUGAL

## Abstract

Accurate DNA quantification is key for downstream application including library preparations for whole genome sequencing (WGS) and the quantification of standards for quantitative PCR. Two commonly used technologies for nucleic acid quantification are based on spectrometry, such as NanoDrop, and fluorometry, such as Qubit. The DS–11+ Series spectrophotometer/fluorometer (DeNovix) is a UV spectrophotometry-based instrument and is a relatively new spectrophotometric method but has not yet been compared to established platforms. Here, we compared three DNA quantification platforms, including two UV spectrophotometry-based techniques (DeNovix and NanoDrop) and one fluorometry-based approach (Qubit). We used genomic prokaryotic DNA extracted from *Streptococcus pneumoniae* using a Roche DNA extraction kit. We also evaluated purity assessment and effect of a single freeze-thaw cycle. Spectrophotometry-based methods reported 3 to 4-fold higher mean DNA concentrations compared to Qubit, both before and after freezing. The ratio of DNA concentrations assessed by spectrophotometry on the one hand, and Qubit on the other hand, was function of the A_260/280_. In case DNA was pure (A_260/280_ between 1.7 and 2.0), the ratio DeNovix or Nanodrop vs. Qubit was close or equal to 2, while this ratio showed an incline for DNA with increasing A_260/280_ values > 2.0. The A_260/280_ and A_260/230_ purity ratios exhibited negligible variation across spectrophotometric methods and freezing conditions. The comparison of DNA concentrations from before and after freezing revealed no statistically significant disparities for each technique. DeNovix exhibited the highest Spearman correlation coefficient (0.999), followed by NanoDrop (0.81), and Qubit (0.77). In summary, there is no difference between DeNovix and NanoDrop in estimated gDNA concentrations of *S*. *pneumoniae*, and the spectrophotometry methods estimated close or equal to 2 times higher concentrations compared to Qubit for pure DNA.

## Introduction

Accurate quantification of DNA extracted from cultured bacteria is essential for downstream applications, including library preparations for whole genome sequencing (WGS) and the quantification of standards for quantitative PCR. Two commonly used technologies for nucleic acid quantification are based on spectrometry and fluorometry [1].

For quantification, spectrometry methods such as NanoDrop and DeNovix rely upon the maximal absorbance of light in the ultraviolet (UV) wavelength range by DNA (and RNA) at 260 nm [1]. The assessment of DNA purity by measuring the ratio of absorbance at 260 and 280 nm (A_260/280_) is a widely used technique, to indicate the presence of, for example, residual peptides and phenolic compounds. These compounds exhibit strong absorption around 280 nm, thereby yielding low A_260/280_ ratios when coexisting with DNA [2–4]. Conventionally, an A_260/280_ ratio ranging from 1.7 to 2.0 is accepted as indicative of pure DNA [4]. Notably, absorbance at 260 nm fails to differentiate between nucleotides, single- and double-stranded (ss-/ds-) DNA, and RNA, potentially leading to erroneous, overestimated purity ratios and dsDNA concentrations [5].

The A_260/230_ ratio represents another parameter for spectrometric DNA quantification. An A_260/230_ ratio approximately between 2.0 and 2.2 typically signifies pure DNA [6, 7]. A_260/230_ ratios lower then 2.0 may signify the presence of 230 nm-absorbing contaminants like proteins, lipids, salts, and phenols [8, 9]. However, this ratio’s reliability is compromised when saline elution buffers are used, as salt also absorbs at 230 nm [10].

Conversely, fluorescence-based DNA quantification techniques use intercalating fluorescent dyes (e.g., Hoechst-33258, PicoGreen) that emit fluorescence upon intercalation in the major groove of dsDNA present [11]. This approach’s advantage lies in the availability of diverse fluorescent dyes targeting specific nucleic acid species, ensuring accurate quantification without the interference of picking up contamination.

Among these technologies, the DS–11+ Series spectrophotometer/fluorometer (DeNovix) is a UV spectrophotometry-based instrument with a combined functionality. However, its performance in DNA quantification remains unexplored compared to established platforms. Thus, our study endeavored to compare the DS–11+ Series spectrophotometer/fluorometer against two benchmark methods: the NanoDrop spectrophotometer (a reference spectrophotometric platform) and the Qubit 4 fluorometer (a fluorometry-based platform). We focused on quantifying prokaryotic DNA and the effects of a single freeze-thaw cycle on DNA quantitation. Importantly, we aimed to provide more insights on the performance of DeNovix and NanoDrop, as spectroscopy methods, compared to Qubit as a fluorometric method, in a routine setting using real-life gDNA extracts from (clinical) prokaryotes, as used daily in a research microbiology laboratory with a clinical background.

## Materials and methods

### Strains and culture conditions

A total of 78 *Streptococcus pneumoniae* strains was selected from the culture collection of the Laboratory Bacteriology Research (LBR; Department of Diagnostic Sciences, Faculty of Medicine and Health Sciences, Ghent University, Belgium) stored at -80°C. *S*. *pneumoniae* strains were cultured for 24 h on tryptic soy agar plates + 5% sheep blood (Becton Dickinson, Erembodegem, Belgium) under aerobic atmosphere supplemented with 5% CO_2_. Identification of the cultured strains was confirmed by means of matrix-assisted laser desorption ionization time-of-flight mass spectrometry (MALDI-TOF MS) as previously described [12].

### DNA extraction

DNA extracts were prepared using the High Pure PCR Template Preparation Kit (Roche Applied Science, Basel, Switzerland). First, bacteria were collected from the culture plates using a sterile 10 μL inoculation loop until the eye of the loop was completely filled or until there were no more colonies on the culture plate to be collected and were then suspended in a 1.7 mL Eppendorf tube containing 200 μL PBS (Lonza, Basel, Switzerland). DNA extraction was performed according to the manufacturer’s instructions with few adaptations to the initial steps: (i) instead of using the recommended 5 μL lysozyme for lysis of the suspended bacteria, 200 μL tissue lysis buffer and 40 μL proteinase K (included in the DNA extraction kit, concentration not disclosed by manufacturer) were added to each PBS suspension; (ii) after this lysis step, the suspensions were supplemented with 2 μL mutanolysin (25 U/μL) and incubated for 15 minutes at 37°C; (iii) after this incubation, 200 μL binding buffer was added to each suspension and mixed immediately with 10 minutes incubation at 70°C. Further steps were performed according to the manufacturer’s protocol. The DNA extracts were placed on ice and were tested with three DNA quantification techniques.

### DNA quantification and purity assessment

#### Quantification platforms

DNA was quantified using three distinct platforms: the DeNovix DS–11+ Series spectrophotometer/fluorometer (DeNovix Inc., Wilmington, NC), the NanoDrop ND-1000 spectrophotometer (Thermo Fisher Scientific, Waltham, MA), and the Qubit 4 fluorometer (Thermo Fisher Scientific). In this manuscript, these platforms are respectively referred to as "DeNovix," "NanoDrop," and "Qubit."

#### DeNovix analysis

The DeNovix platform is multifunctional and allows quantitation of proteins and nucleic acids, the latter can be quantified using spectrophotometry (through microvolume quantitation) and fluorometry. In this study, the spectrophotometric method was used to quantify dsDNA and is characterized by a dynamic range of 0.75–37500 ng/μL (dsDNA) and a pathlength, to calculate the absorbances, of 10 mm (transformed).

For the DeNovix platform, a blank measurement of 1 μL elution buffer from the High Pure PCR Template Preparation Kit was carried out using the "dsDNA" program, prior to the analysis of 1 μL of the bacterial DNA extracts. Each DNA extract was measured twice for technical replication. Resulting data, including DNA concentrations, A_260/280_ and A_260/230_ ratios, were exported in.csv format.

Between measurements, the analytic column and lid of the instrument were cleaned using Kimtech Science precision wipes (Kimberly-Clark Professional, Roswell, NM).

#### NanoDrop analysis

Similar to the DeNovix platform, NanoDrop quantification of dsDNA is based on microvolume quantitation with a dynamic range of 2–3700 ng/μL (dsDNA) and pathlength, to calculate the absorbances, of 10 mm (transformed).

On the NanoDrop spectrophotometer, a first blank measurement using 1 μL ultrapure water was measured, followed by a second blank of 1 μL elution buffer from the High Pure PCR Template Preparation Kit prior to quantifying 1 μL of the bacterial DNA extracts twice (i.e. two technical replicates). Measurements were recorded in the NanoDrop ND-1000 3.3 software. Output data, i.e., DNA concentrations and absorbance ratios, were exported as.txt files for subsequent analysis.

Between measurements, the analytic column and lid of the instrument were cleaned using Kimtech Science precision wipes (Kimberly-Clark Professional).

#### Qubit analysis

Utilizing the 1X dsDNA high sensitivity kit (Thermo Fisher Scientific) with a dynamic range of 0.005–120 ng/μL (dsDNA) and pathlength of 10 mm for absorbance calculation, DNA samples were prepared according to the manufacturer’s guidelines for the Qubit fluorometer. Prior to measuring 1 μL from each DNA extract twice, the control solutions included in the kit were measured for quality control. The resulting data were exported in.csv format.

### Quantification timeline and sample handling

DNA concentrations for all extracts were determined immediately post-extraction (T_1_) and after one week of storage at -20°C (T_2_). Throughout measurements, the DNA extracts were maintained on ice.

### Data analysis

The DNA concentrations were expressed in ng/μL. The average DNA concentrations, A_260/280_ and A_260/230_ ratios from two technical replicates were computed and used in all subsequent analyses.

All statistical analyses were executed using R. Medians and ranges of DNA concentrations, A_260/230_, and A_260/280_ ratios were documented for each platform and time point. To identify significant differences in mean in DNA concentration, A_260/230_, and A_260/280_ ratios among DNA quantitation techniques and timepoints, the Wilcoxon signed-rank test was used. The presented violin-strip plots specifically depict Wilcoxon significance testing outcomes for notable changes corresponding to p-values<0.001.

Scatter plots and violin-strip plots were generated through modeling in Python to visually elucidate correlations and statistical characteristics of the compared parameters.

Regression analysis of scatter plot data was carried out by means of a statistical linear regression model with an origin intercept. This yielded a regression line, the multiple R-squared value (R^2^), and p-value for the regression model. Additionally, a line of equality (y = x) was incorporated for reference.

The Spearman rank correlation (R_S_) and corresponding p-value were computed to ascertain the relationships between datasets, as described above. P-values below 0.001 were indicative of significance.

## Results

### DNA quantification with three different techniques

**Fig 1:**
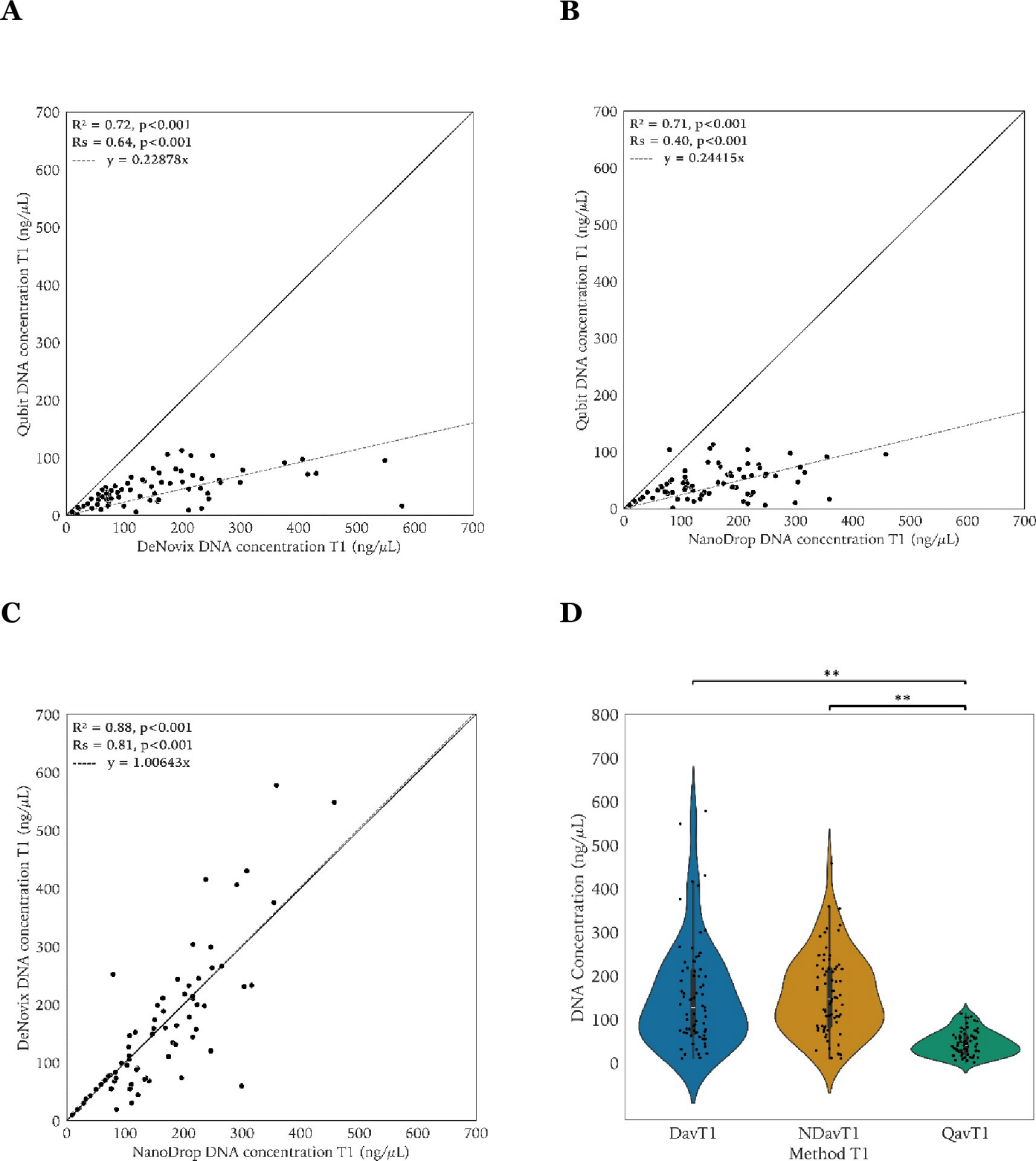
Scatter and violin-strip plots of the DNA concentrations at T_1_. Scatter plot of Qubit−DeNovix (**Panel A)**, Qubit−NanoDrop (**Panel B**) and DeNovix−NanoDrop (**Panel C**). The multiple R-squared (R^2^), Spearman correlation (R_S_), and corresponding p-values are provided. The line of equality is presented as a diagonal line and the dashed line corresponds to the linear regression model with formula as indicated. Violin-strip plots of the DNA concentrations at T_1_ by DeNovix, NanoDrop, and Qubit (**Panel D**). Statistically significant differences (Wilcoxon test, p<0.001) are marked with **. Dav: DeNovix mean DNA concentrations, NDav: NanoDrop mean DNA concentrations, Qav: Qubit mean DNA concentrations,T_1_: prior to freeze storing.

The scatter plots and box plots of the DNA concentrations measured at T_1_ on the three platforms are shown in **Fig 1**. The median DNA concentrations were 40 ng/μL (range 0.98–113) for Qubit, 146.72 ng/μL (range 9.67–457.58) for NanoDrop, and 126.93 ng/μL (10.01–577.98) for DeNovix. The Qubit−NanoDrop and Qubit−DeNovix differences in mean were statistically significant. The R^2^ of the linear regression models were 0.72 (Qubit−DeNovix), 0.71 (Qubit−NanoDrop) and 0.88 (DeNovix−NanoDrop), and all were statistically significant (p<0.001). Spearman rank correlations were 0.64 (Qubit−DeNovix), 0.40 (Qubit−NanoDrop) and 0.81 (DeNovix−NanoDrop), all statistically significant (p<0.001). The slope coefficients of the regression model were 0.23 (Qubit−DeNovix) and 0.24 (Qubit−NanoDrop), suggesting that the DNA concentrations determined by spectrophotometric methods were about 3 to 4 times higher compared to Qubit.

**Fig 2:**
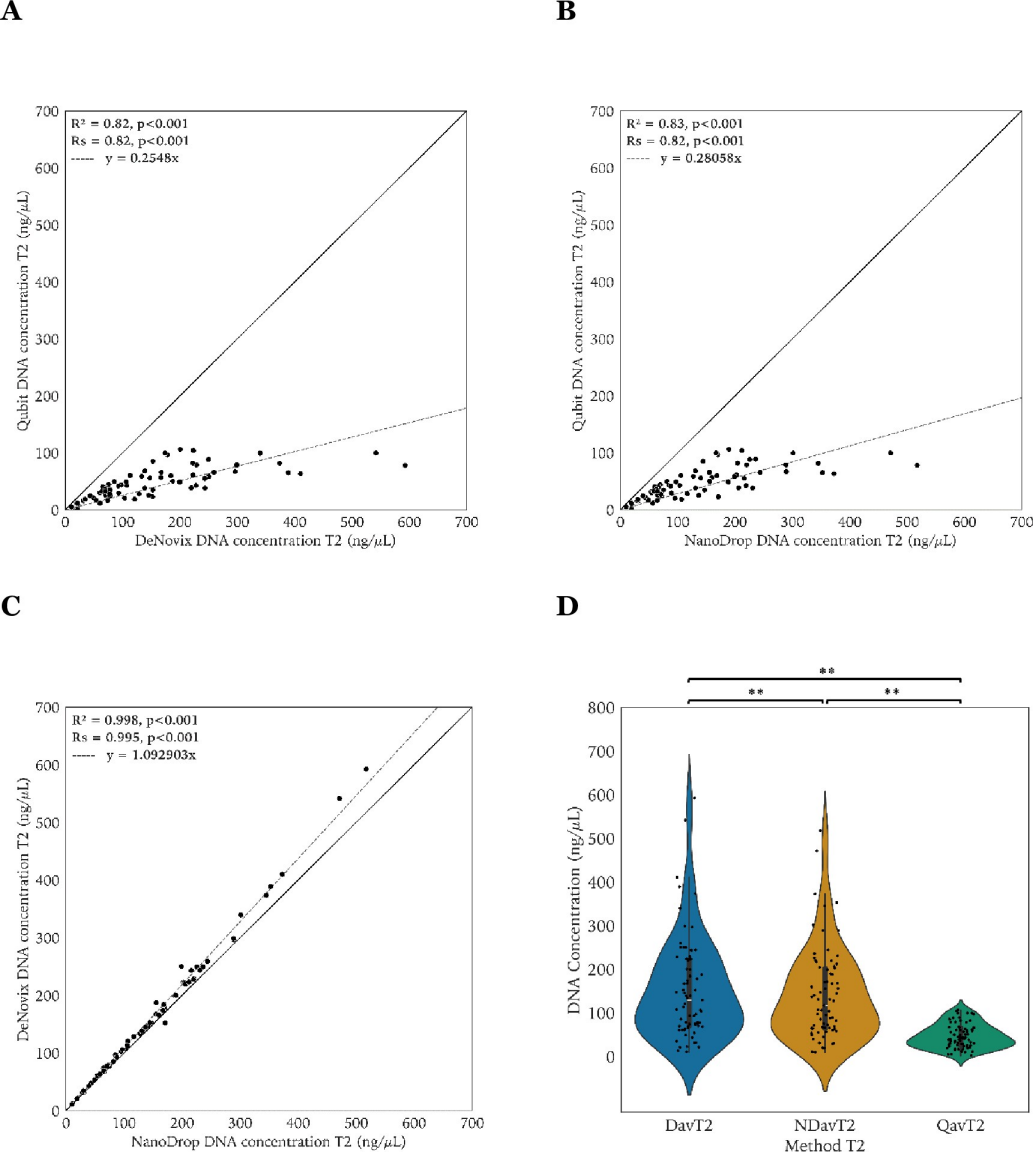
Scatter and violin-strip plots of the DNA concentrations at T_2_. Scatter plot of Qubit−DeNovix (**Panel A)**, Qubit−NanoDrop (**Panel B**) and DeNovix−NanoDrop (**Panel C**). The multiple R-squared (R^2^), Spearman correlation (R_S_), and corresponding p-values are provided. The line of equality is presented as a diagonal line and the dashed line corresponds to the linear regression model with formula as indicated. Violin-strip plots of the DNA concentrations at T_2_ by DeNovix, NanoDrop, and Qubit (**Panel D**). Statistically significant differences (Wilcoxon test, p<0.001) are marked with **. Dav: DeNovix mean DNA concentrations, NDav: NanoDrop mean DNA concentrations, Qav: Qubit mean DNA concentrations, T_2_: after freeze storing.

The scatter plots and box plots of the DNA concentrations measured at T_2_ are shown in **Fig 2**. The median DNA concentrations were 41.90 ng/μL (range 1.42–106) for Qubit, 116.55 ng/μL (range 9.83–517.36) for NanoDrop, and 128.49 ng/μL (10.76–592.52) for DeNovix (**Fig 2**). The R^2^ of the linear regression models were 0.82 (Qubit−DeNovix), 0.83 (Qubit−NanoDrop) and 0.998 (DeNovix−NanoDrop), and all were statistically significant (p<0.001). Spearman rank correlations were 0.82 (Qubit−DeNovix), 0.82 (Qubit−NanoDrop) and 0.995 (DeNovix−NanoDrop), all statistically significant (p<0.001). The slope coefficient of the regression model was 0.25 (Qubit−DeNovix) and 0.28 (Qubit−NanoDrop), suggesting that the DNA concentrations determined by spectrophotometric methods were again about 3–4 times higher compared to Qubit.

All measurements were within the dynamic range of the DeNovix and NanoDrop, however, 4 and 5 out of 78 measurements were outside the dynamic range of Qubit (>120 ng/μL dsDNA), before and after freezing, respectively, and were excluded from the analyses.

**Fig 3:**
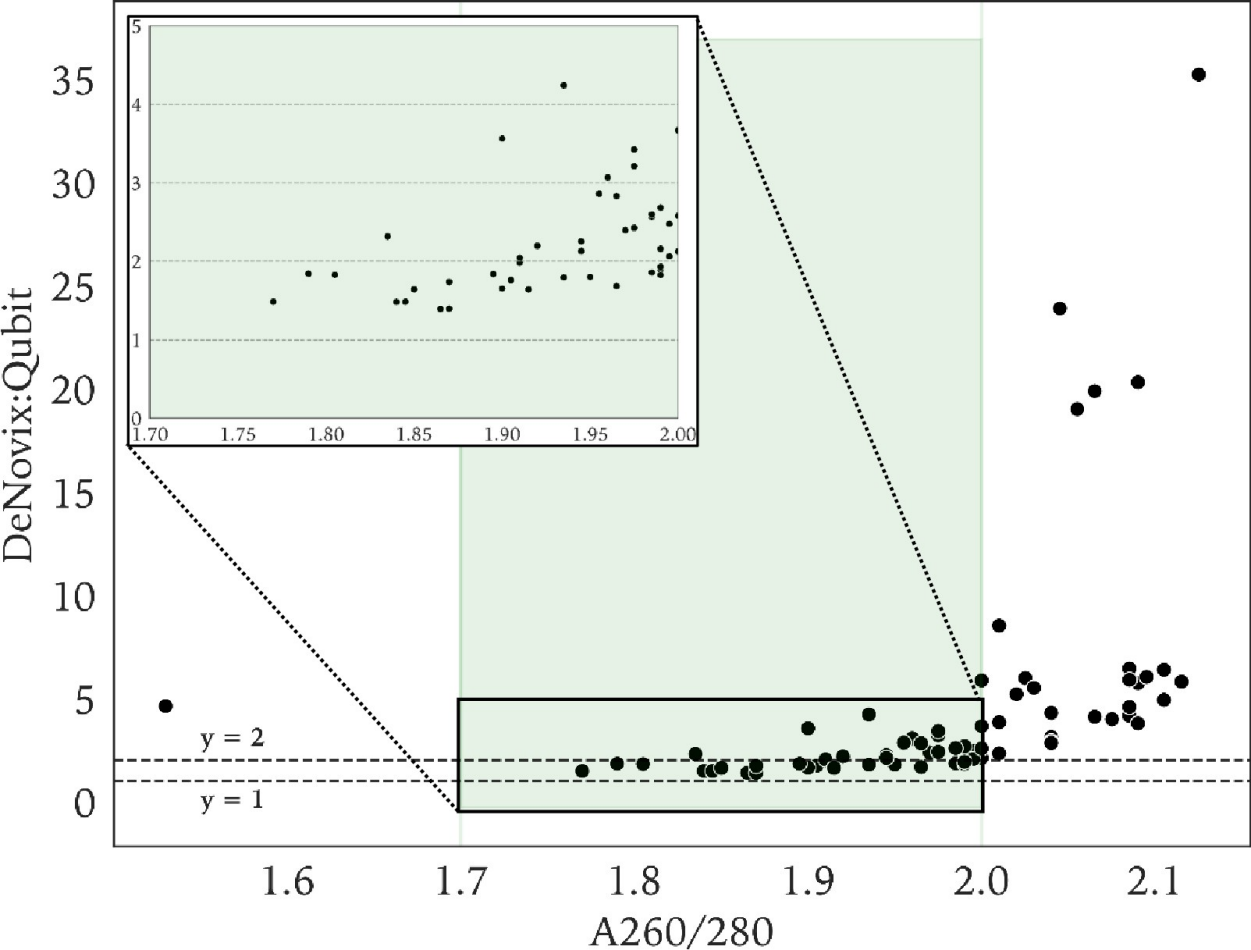
Scatter plot of the ratio of mean DNA concentrations measured by DeNovix and Qubit in function of the DeNovix A_260/280_ purity ratio measured at T_1_. The plot area between 1.7 and 2.0 on the x-axis is highlighted and depicts the interval where DNA is regarded as pure. A more detailed view of this area is shown as an inset plot.

The correlation between the ratio of DeNovix/Qubit DNA quantification on the one hand, and the A_260/280_ ratio on the other hand are shown in **Fig 3**. Most of the DNA concentrations with an A_260/280_ between 1.7 and 2.0, considered as ‘pure’ DNA, had a DeNovix/Qubit ratio close or equal to 2 (median = 2.06, range 1.39–5.88). In contrast, when A_260/280_ exceeded 2.0, ratios for DeNovix/Qubit increased in function of the A_260/280_. For the A_260/230_ ratio, no such observations were made (not shown).

Additionally, the comparison of DNA concentrations measured by Qubit and DeNovix at T_1_ and T_2_ where the DeNovix A_260/280_ ratio at T_1_ was between 1.7−2.0 (considered as ‘pure’ DNA), is shown in **S1 Fig**. The R^2^ of the linear regression models were 0.92 (Qubit−DeNovix at T_1_) and 0.93 (Qubit−DeNovix at T_2_), and all were statistically significant (p<0.001). Spearman rank correlations were 0.86 (Qubit−DeNovix at T_1_) and 0.90 (Qubit−DeNovix at T_2_), all statistically significant (p<0.001). The slope coefficient of the regression model was 0.43 (Qubit−DeNovix at T_1_) and 0.41 (Qubit−DeNovix at T_2_).

### DNA purity assessment

**Fig 4:**
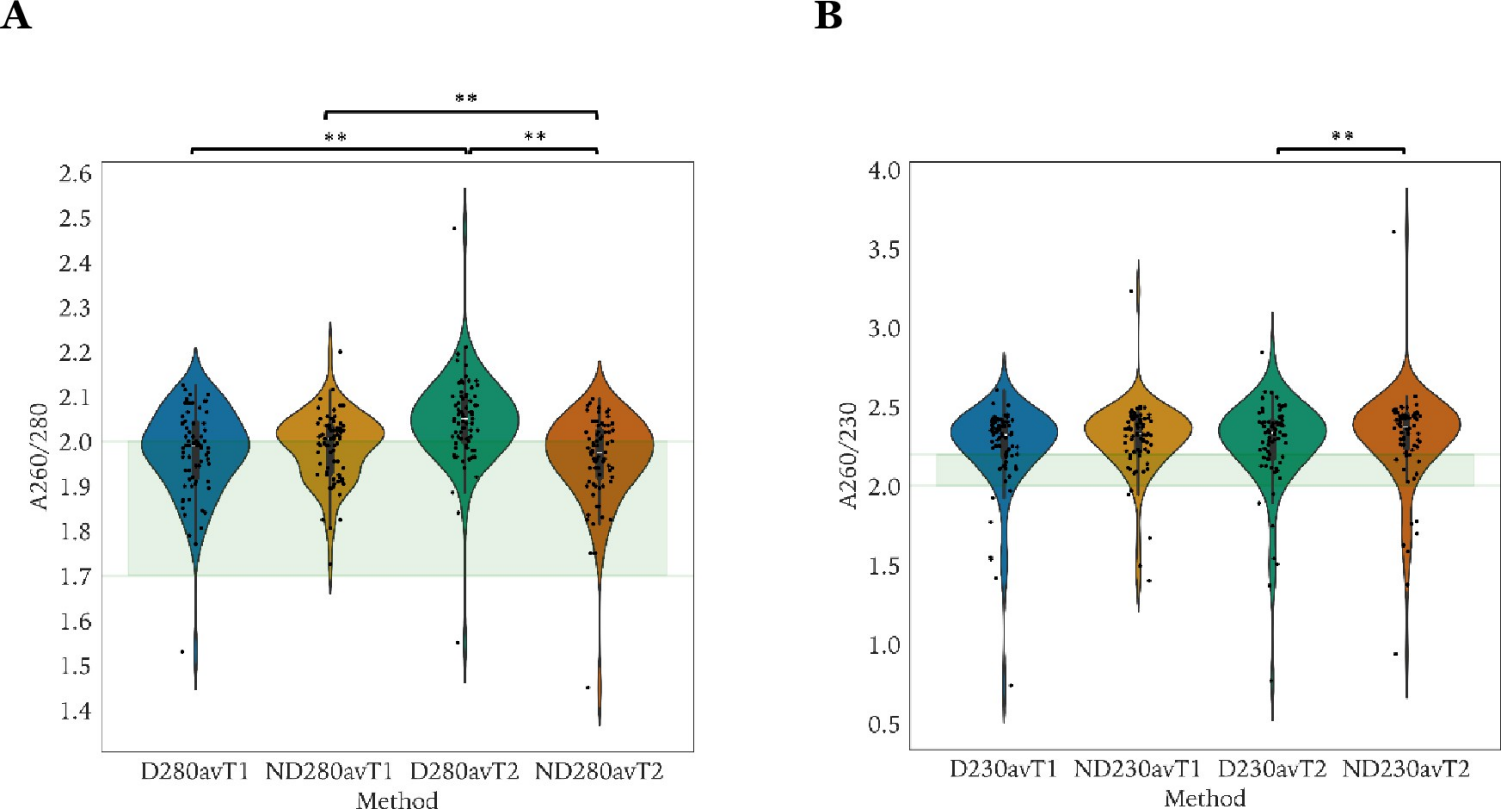
Violin-strip plots of the A_260/280_ and A_260/230_ ratio. The A_260/280_ and A_260/230_ ratio for each timepoint and quantification platform is depicted in **Panel A** and **Panel B**, respectively. For each ratio, the proposed reference interval is indicated where contamination is minimal (A_260/280_: [1.7,2.0], A_260/230_: [2.0,2.2]). Statistically significant differences (Wilcoxon test, p<0.001) are marked with **. D280av: DeNovix mean values A_260/280_, ND280av: NanoDrop mean values A_260/280_. D230av: DeNovix mean values A_260/230_, ND230av: NanoDrop mean values A_260/230_. T_1_: prior to freeze storing, T_2_: after freeze storing.

The A_260/280_ and A_260/230_ ratios for T_1_ and T_2_ are shown in **Fig 4**. The median A_260/280_ ratio for DeNovix and NanoDrop at T_1_ was 1.99 (range 1.53–2.13) and 2.01 (range 1.73–2.20) (**Fig 4A**), respectively, and at T_2_, 2.05 (range 1.55–2.48) and 1.98 (range 1.45–2.10), respectively. The median A_260/230_ ratio for DeNovix and NanoDrop at T_1_ was 2.33 (range 0.74–2.61) and 2.33 (range 1.40–3.23), respectively, and at T_2_, 2.34 (range 0.77–2.85) and 2.38 (range 0.94–3.61) (**Fig 4B**), respectively. Significant differences were found for A_260/280_ DeNovix−NanoDrop at T_2_, A_260/280_ DeNovix T_1_−DeNovix T_2_, A_260/280_ NanoDrop T_1_−NanoDrop T_2_, A_260/230_ DeNovix−NanoDrop at T_2_.

**Fig 5:**
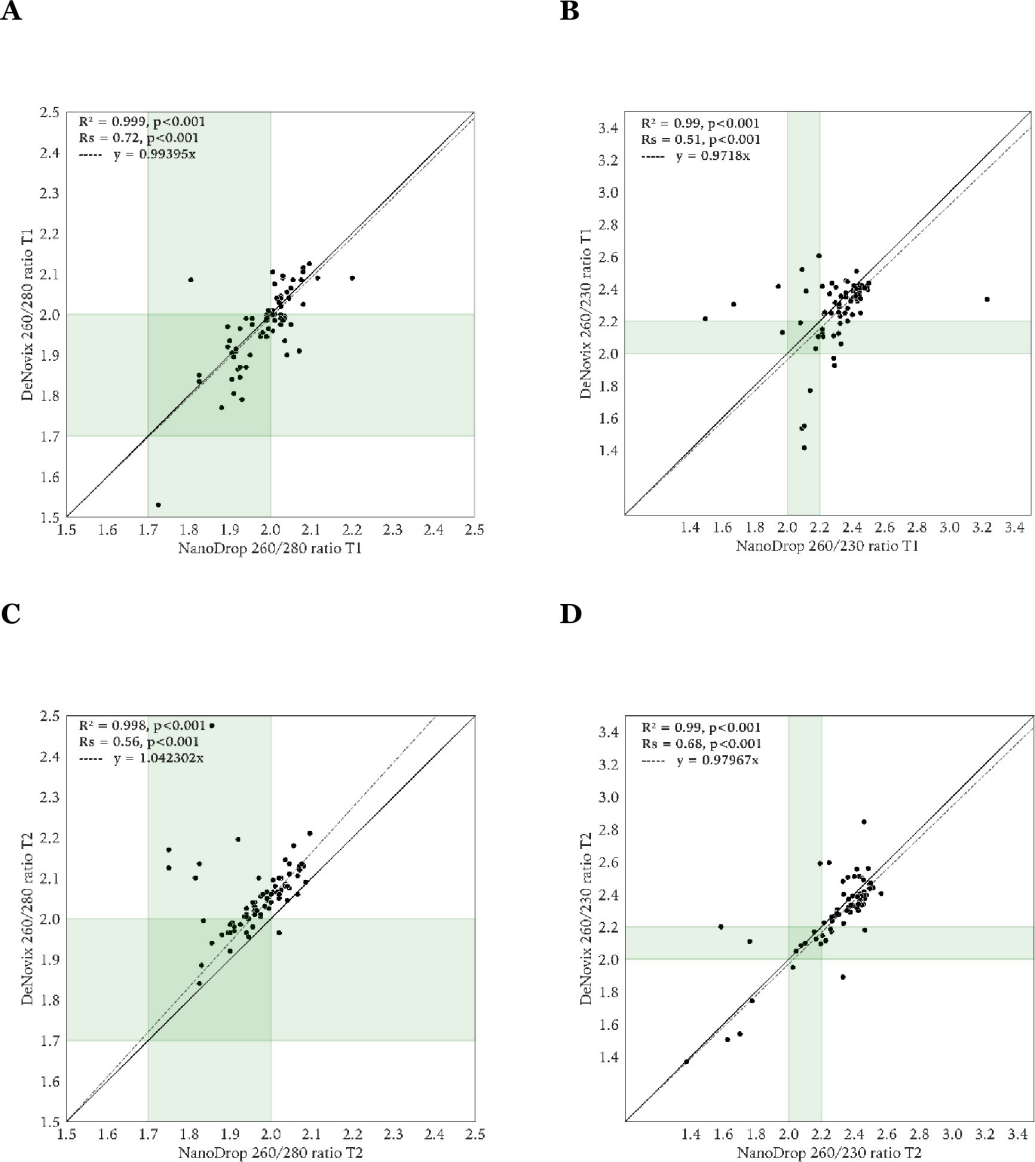
Scatter plots of the A_260/280_ and A_260/230_ ratio for T_1_ (**Panel A and B**) and T_2_ (**Panel C and D**) between DeNovix and NanoDrop. The multiple R-squared (R^2^), Spearman correlation (R_S_), and corresponding p-values are provided. The line of equality is presented as a diagonal line. The dashed line corresponds to the linear regression model with formula as indicated. For each ratio, the proposed reference interval is indicated where DNA is regarded as pure (A_260/280_: [1.7,2.0], A_260/230_: [2.0,2.2]). T_1_, prior to freeze storing; T_2_, after freeze storing.

The scatter plot analysis of the A_260/280_ ratio and A_260/230_ ratio between the DeNovix and NanoDrop measurements at T_1_ and T_2_ is shown in **Fig 5**. For the DeNovix−NanoDrop comparison of the purity ratios at T_1_, the R^2^ of the linear regression model for the A_260/280_ ratio was 0.999 (**Fig 5A**), and for the A_260/230_ ratio 0.99 (**Fig 5B**). At T_2_ these were 0.998 (**Fig 5C**) and 0.99 (**Fig 5D**), respectively. The Spearman rank correlation at T_1_ for the A_260/280_ ratio was 0.72 (**Fig 5A**) and 0.51 for the A_260/230_ ratio (**Fig 5B**). At T_2_ these were 0.56 (**Fig 5C**) and 0.68 (**Fig 5D**), respectively. All were statistically significant (p<0.001).

### Influence of one freeze-thaw cycle on DNA quantification and purity

The scatter plots of the DNA concentrations measured at T_1_ and T_2_ are shown in **S2 Fig**. The R^2^ (R_S_) values were 0.999 (0.999) (DeNovix), 0.89 (0.81) (NanoDrop) and 0.89 (0.77) (Qubit), all p<0.001. No significant differences in mean DNA concentration between T_1_ and T_2_ were observed.

The scatter plots of the A_**260/280**_ ratio and A_**260/230**_ comparison between T_**1**_ and T_**2**_ are shown in **S3A and S3B Fig** for the DeNovix and NanoDrop, respectively.

## Discussion

We are the first to compare the DeNovix platform to other established platforms (NanoDrop and Qubit) for its performance in DNA quantification and/or purity assessment. We focused on prokaryotic gDNA derived from *S*. *pneumoniae*. Additionally, we investigated the evaluation of these techniques for their susceptibility to variations induced by a single freeze-thaw cycle of DNA extracts.

### DeNovix and NanoDrop performed equally well

The DNA concentrations, A_260/280_ and A_260/230_ values exhibited a high degree of correlation between the spectrophotometry-based techniques DeNovix and NanoDrop (with R_S_ ranging from 0.81 to 0.995). This strong correlation was observed both before and after freezing, with regression equations closely approximating the identity line (y = x). Prior to freezing, there was no statistically significant difference in the median DNA concentrations measured by the DeNovix and NanoDrop instruments (127 ng/μl vs. 147 ng/μl, respectively), whereas post-freeze, this difference was statistically significant but considered minimal (128 ng/μl vs. 117 ng/μl, respectively). Notably, both the DeNovix and NanoDrop instruments provided very similar mean A_260/280_ and A_260/230_ values at both time points. Overall, both spectrophotometric methods yielded highly consistent results for DNA concentration and purity, with minimal discrepancies.

### Spectrophotometric methods estimated higher mean DNA concentrations compared to Qubit

DeNovix and NanoDrop reported mean DNA concentrations of about 3–4 times higher compared to Qubit, both before and after freezing, but only about 2.5 times higher when only ‘pure’ DNA extracts (A_260/280_ ratio between 1.7 and 2) were considered.

Because of the lack of a gold standard to assess DNA concentrations, it is unclear to what extent the spectrophotometric methods and Qubit in our study are either under- or overestimating. Raw data-analysis did not show baseline absorbance above 310 nm which demonstrates that there is no indication of aggregates or foreign large particles which could cause light scattering that would add to the absorbance, increasing with decreasing wavelength, and overestimating the absorbance readings. On the one hand, in our study, the higher DNA concentrations reported by DeNovix and NanoDrop might by (partially) overestimated and caused by contaminants, such as proteins and salts, as documented in previous studies [8, 9, 13–15]. In this regard, we observed that for DNA extracts with A_260/280_ values between 1.7 and 2.0, regarded as pure DNA, the concentration ratios DeNovix/Qubit and NanoDrop/Qubit were rather close or equal to 2. For A_260/280_ values > 2.0, these ratios were higher than 2 and positively associated with A_260/280_. Data from gDNA (A_260/280_ between 1.7 and 2.0) from 50 *Escherichia coli* strains quantified with DeNovix and Qubit showed a mean ratio of one (Versmessen *et al*., Average nucleotide identity and digital DNA-DNA hybridization analysis following PromethION nanopore-based whole genome sequencing allows for accurate prokaryotic typing, under review). The reason for the discrepancy with current findings is not clear.

An additional factor possibly contributing to overestimations by spectrophotometric methods stems from leaching of compounds from plastic tubes during storage [16]. These leachates are composed of a variety of additives, which are added by the manufacturer to the plastic to maintain its integrity, and their breakdown products which diffuse from the plastic tubes during handling of the tubes (e.g. processing and heating) as well as due to ageing of the plastic. The leachates can contain chromophores that absorb UV light at 230 nm and 260 nm, wavelengths also absorbed by proteins and nucleic acids, respectively. It has been shown that these leachates can lead to an overestimation of DNA concentrations of up to 55% for the most commonly used tubes (containing polypropylene) and up to 300% for others [16].

On the other hand, in our study, the lower Qubit DNA concentrations might be (partially) underestimated compared to DeNovix and NanoDrop. Fluorometry-based DNA quantification methods target specific DNA molecules, such as dsDNA, and are regarded immune to interference by contaminants [8, 9, 13–15]. However, previous studies have highlighted Qubit’s sensitivity to dsDNA fragmentation and denaturation due to laboratory manipulations or freeze-thaw cycles [14, 17–19]. Repeated freeze-thaw cycles and long-term storage are the most important cause of DNA degradation, such as fragmentation, as well as continued chemical and physical reactions in frozen samples, in contrary to the widespread conviction that deep frozen samples are not subjected to any changes [20–22]. Previous research further indicates that Qubit tends to underestimate the dsDNA concentration when high molecular weight DNA or fragments of <23 kbps are analyzed, and when eluates containing salt solutions are involved [14]. In our study, we did not assess the fragment lengths of our DNA extracts. However, gDNA from 50 *Escherichia coli* strains extracted using the same Roche kit in our lab, showed that most DNA extracts had a size around 20 kbps (80%), and the others 10 kbps or lower (Versmessen *et al*., Average nucleotide identity and digital DNA-DNA hybridization analysis following PromethION nanopore-based whole genome sequencing allows for accurate prokaryotic typing, under review).

There are few studies comparing different quantitative methods for nucleic acid species with varying or often conflictive results. The tendency of DNA concentrations reported to be higher using NanoDrop vs. Qubit was previously described [15, 23]. While some studies favor Qubit over NanoDrop [24], other studies report spectrophotometry to be more concordant to a reference method compared to fluorometry and qPCR [24, 25]. Simbolo *et al*. reported that for high molecular weight DNA derived from histopathological samples, NanoDrop DNA concentrations were higher than those from the Qubit but consistent with qPCR dsDNA quantification, however, for degraded DNA, Qubit was more consistent with qPCR [15]. Both factors, degradation and absence of information about contamination of the samples, presented the risk of NGS library failure. Therefore, it was suggested that only the combination of both methods provide correct qualification of DNA samples [15]. In terms of accuracy, the accuracy of Qubit assays were reported to be more reliable than NanoDrop, nonetheless, qPCR stands out as the optimal method for DNA quantification but is time- and cost-expensive and is therefore not suited for every lab [15, 23].

### A single freeze/thaw cycle did not affect DNA concentrations and purity parameter values

The A_260/280_ and A_260/230_ ratios, indicating purity, exhibited negligible variation across spectrophotometric methods and freezing conditions. The comparison of DNA concentrations from before and after freezing revealed no statistically significant disparities for each technique. DeNovix exhibited the highest Spearman correlation coefficient (0.999), followed by NanoDrop (0.81), and Qubit (0.77). These findings underscore the stability of reported DNA concentrations (and purity ratios) measured by spectrophotometry and Qubit upon a single freeze-thaw cycle.

### Study limitations and future perspectives

Our study was limited by the use of DNA extracts from only a single species and a single extraction method, and the findings may not necessarily generalize to other bacterial species and extraction methods. While our study aimed to provide a comparative analysis of the performance of spectrophotometric and fluorometric methods in quantifying genomic DNA, our investigation did not include experimental attempts to elucidate the observed discrepancies in results. Future studies could incorporate experimental designs aimed at uncovering the underlying factors contributing to the observed differences in DNA quantification between the methods evaluated in this study. Further factors to consider are the cost-time trade-off. NanoDrop and DeNovix allow rapid sample processing (≤1 minute) at a low cost, as opposed to Qubit with lengthier processing times (>5 minutes) and higher costs per sample [15].

## Conclusion

To quantify gDNA from S. pneumoniae and assess DNA purity, DeNovix and NanoDrop performed equally well. DeNovix and NanoDrop DNA mean concentrations were about 3 to 4 times higher compared to Qubit, but in case the DNA was pure, the ratio DeNovix (or NanoDrop)/Qubit was close or equal to 2. A single freeze-thaw cycle had no effect on quantification and purity assessment for all platforms.

## Supporting information

S1 Fig Scatter plots comparing the DNA concentrations measured by Qubit and DeNovix at T1 (left) and T2 (right), and only for DNA concentration measurements where DeNovix T1 A260/280 was between 1.7–2.0. The multiple R-squared (R2), Spearman correlation (RS), and corresponding p-values are provided. The line of equality is presented as a diagonal line. The dashed line corresponds to the linear regression model with formula as indicated. T1, timepoint 1 (before freeze storing); T2, timepoint 2 (after freeze storing).
(DOCX)

S2 Fig Scatter plots of the DNA concentrations compared between T1 and T2 of DeNovix (Panel A), NanoDrop (Panel B) and Qubit (Panel C). The multiple R-squared (R2), Spearman correlation (RS), and corresponding p-values are provided. The line of equality is presented as a diagonal line. The dashed line corresponds to the linear regression model with formula as indicated. T1, timepoint 1 (before freeze storing); T2, timepoint 2 (after freeze storing).
(DOCX)

S3 Fig Scatter plots of the A260/280 ratio (Panel A) and A260/230 ratio (Panel B) compared between timepoints T1 and T2 (before and after freezing) on the DeNovix (left) and NanoDrop (right). The multiple R-squared (R2), Spearman correlation (RS), and corresponding p-values are provided. The line of equality is presented as a diagonal line. The dashed line corresponds to the linear regression model with formula as indicated. For each ratio, the proposed reference interval is indicated where DNA is regarded as pure (A260/280: [1.7,2.0], A260/230: [2.0,2.2]). T1, timepoint 1 (before freeze storing); T2, timepoint 2 (after freeze storing).
(DOCX)
