# Supplementary material for: Comparison of DeNovix, NanoDrop and Qubit for DNA quantification and impurity detection of bacterial DNA extracts
Source: PLoS One. 2024 Jun 17;19(6):e0305650. doi: 10.1371/journal.pone.0305650 (PMC11182499; doi:10.1371/journal.pone.0305650)
Supplement: S1 Fig — Scatter plots comparing the DNA concentrations measured by Qubit and DeNovix at T1 (left) and T2 (right), and only for DNA concentration measurements where DeNovix T1 A260/280 was between 1.7–2.0. The multiple R-squared (R2), Spearman correlation (RS), and corresponding p-values are provided. The line of equality is presented as a diagonal line. The dashed line corresponds to the linear regression model with formula as indicated. T1, timepoint 1 (before freeze storing); T2, timepoint 2 (after freeze storing). (DOCX) [file pone.0305650.s001.docx]

| **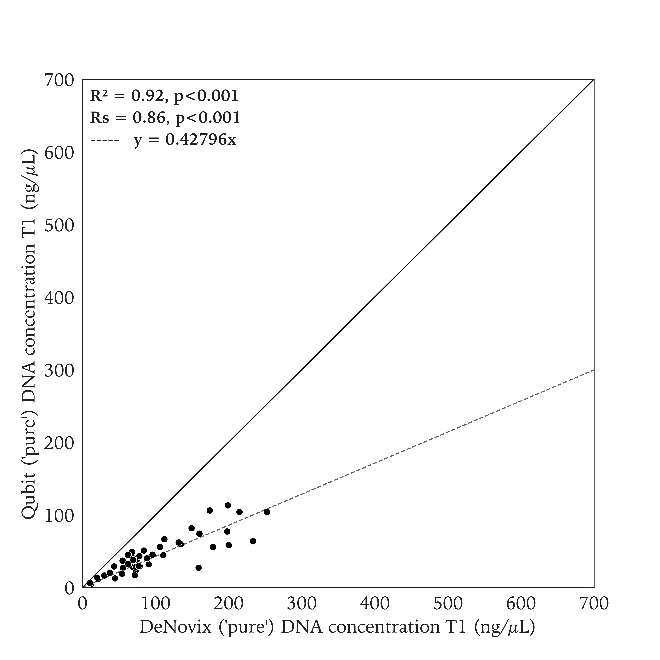** | **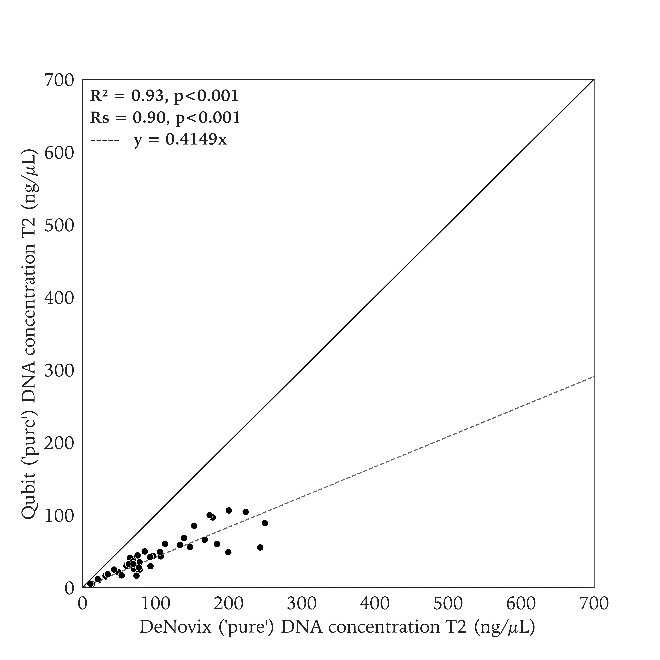** |
| --- | --- |
| **SI 1. Scatter plots comparing the DNA concentrations measured by Qubit and DeNovix at T_1_ (left) and T_2_ (right), and only for DNA concentration measurements where DeNovix T_1_ A_260/280_ was between 1.7−2.0.** The multiple R-squared (R^2^), Spearman correlation (R_S_), and corresponding p-values are provided. The line of equality is presented as a diagonal line. The dashed line corresponds to the linear regression model with formula as indicated. T_1_, timepoint 1 (before freeze storing); T_2_, timepoint 2 (after freeze storing). | |
