# Supplementary material for: Comparison of DeNovix, NanoDrop and Qubit for DNA quantification and impurity detection of bacterial DNA extracts
Source: PLoS One. 2024 Jun 17;19(6):e0305650. doi: 10.1371/journal.pone.0305650 (PMC11182499; doi:10.1371/journal.pone.0305650)
Supplement: S2 Fig — Scatter plots of the DNA concentrations compared between T1 and T2 of DeNovix (Panel A), NanoDrop (Panel B) and Qubit (Panel C). The multiple R-squared (R2), Spearman correlation (RS), and corresponding p-values are provided. The line of equality is presented as a diagonal line. The dashed line corresponds to the linear regression model with formula as indicated. T1, timepoint 1 (before freeze storing); T2, timepoint 2 (after freeze storing). (DOCX) [file pone.0305650.s002.docx]

| **A** | **B** |
| --- | --- |
| **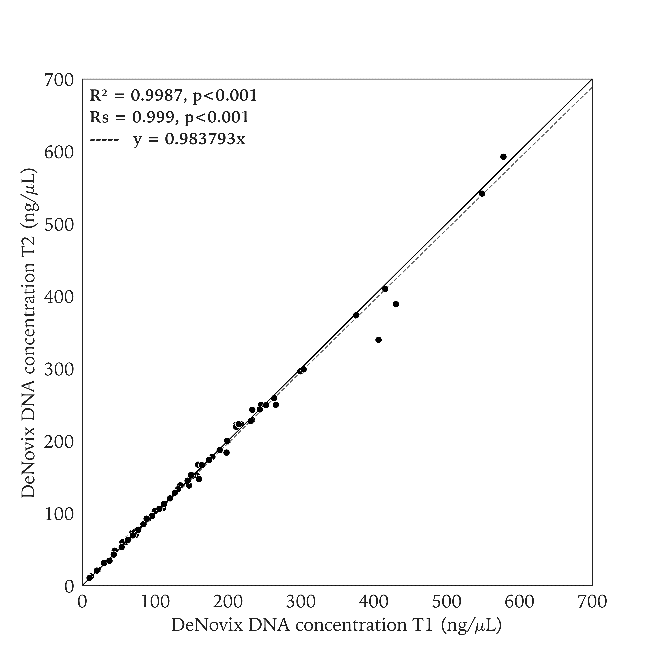** | **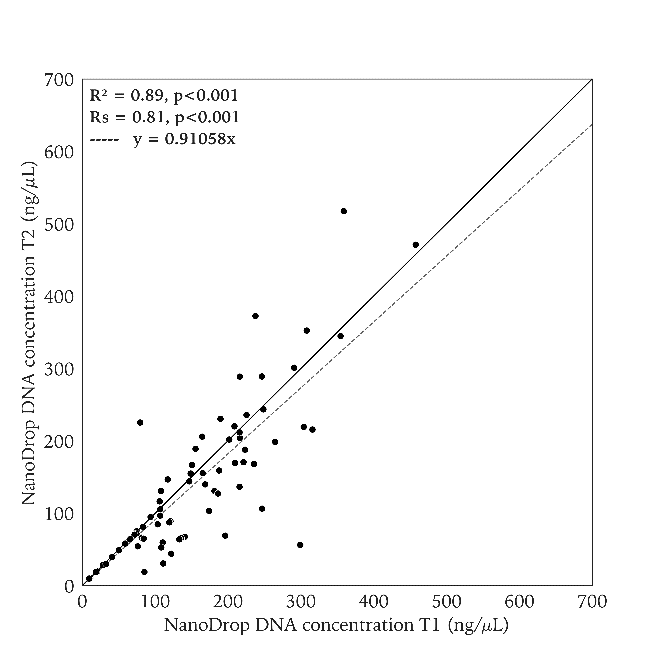** |
| **C** |  |
| **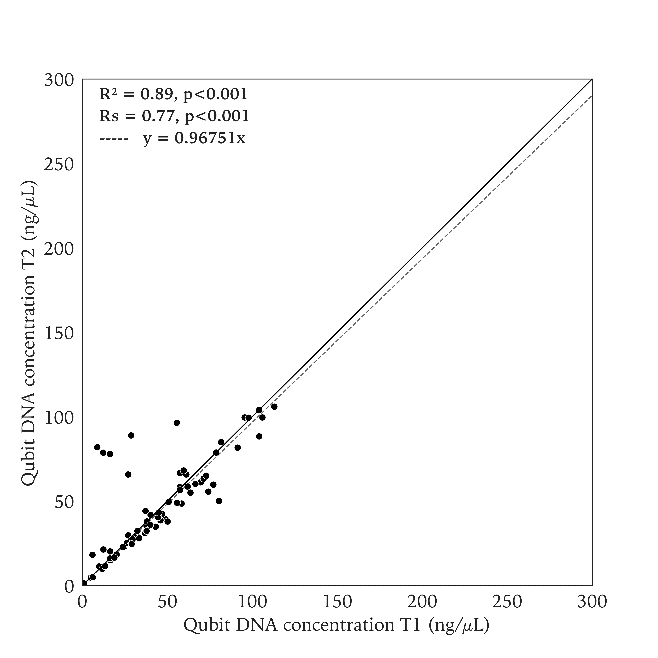** |  |
| **SI 2.** Scatter plots of the DNA concentrations compared between T_1_ and T_2_ of DeNovix (**Panel A),** NanoDrop (**Panel B**) and Qubit (**Panel C**). The multiple R-squared (R^2^), Spearman correlation (R_S_), and corresponding p-values are provided. The line of equality is presented as a diagonal line. The dashed line corresponds to the linear regression model with formula as indicated. T_1_, timepoint 1 (before freeze storing); T_2_, timepoint 2 (after freeze storing). | |
