# Supplementary material for: Comparison of DeNovix, NanoDrop and Qubit for DNA quantification and impurity detection of bacterial DNA extracts
Source: PLoS One. 2024 Jun 17;19(6):e0305650. doi: 10.1371/journal.pone.0305650 (PMC11182499; doi:10.1371/journal.pone.0305650)
Supplement: S3 Fig — Scatter plots of the A260/280 ratio (Panel A) and A260/230 ratio (Panel B) compared between timepoints T1 and T2 (before and after freezing) on the DeNovix (left) and NanoDrop (right). The multiple R-squared (R2), Spearman correlation (RS), and corresponding p-values are provided. The line of equality is presented as a diagonal line. The dashed line corresponds to the linear regression model with formula as indicated. For each ratio, the proposed reference interval is indicated where DNA is regarded as pure (A260/280: [1.7,2.0], A260/230: [2.0,2.2]). T1, timepoint 1 (before freeze storing); T2, timepoint 2 (after freeze storing). (DOCX) [file pone.0305650.s003.docx]

| **A** | |
| --- | --- |
| 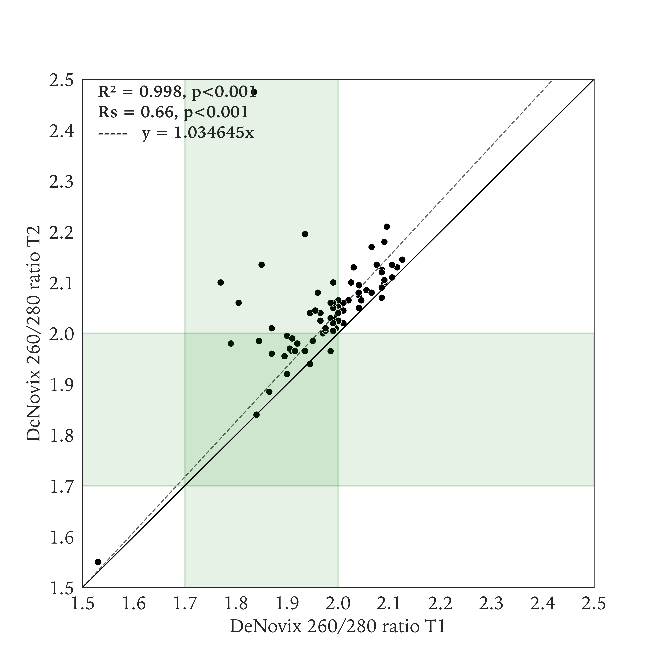 | 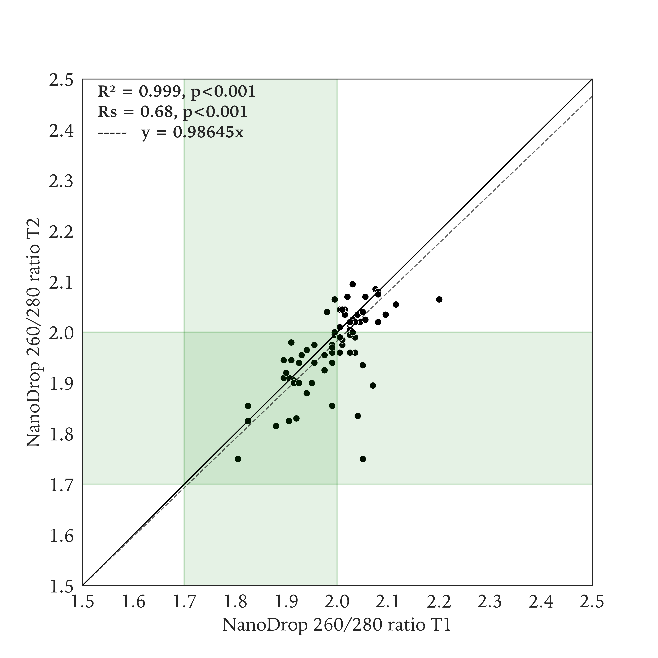 |
| **B** | |
| 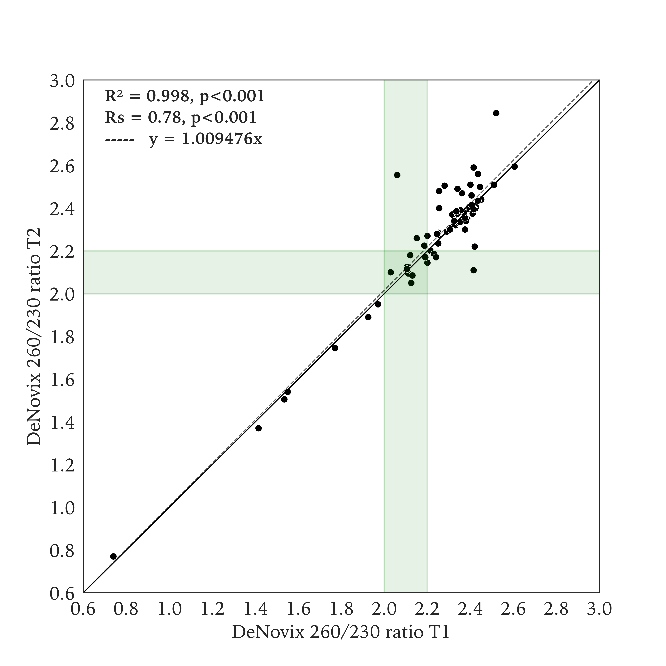 | 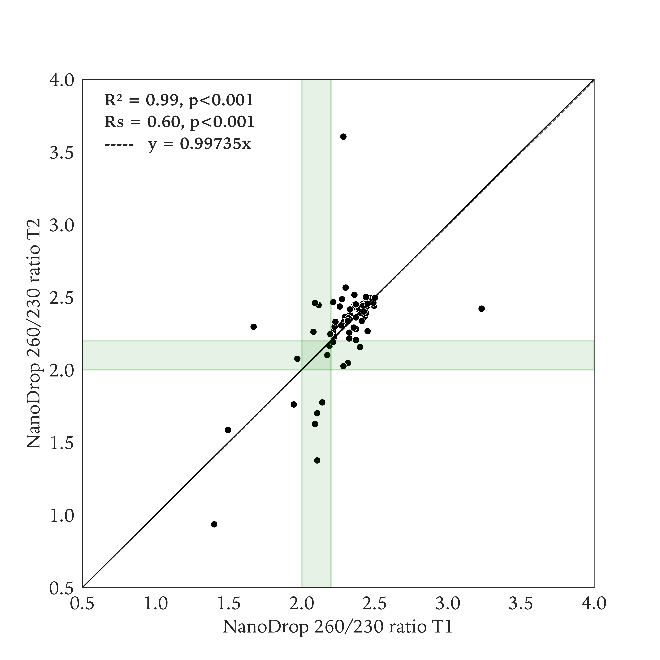 |
| **SI 3.** Scatter plots of the A_260/280_ ratio (**Panel A**) and A_260/230_ ratio (**Panel B**) compared between timepoints T_1_ and T_2_ (before and after freezing) on the DeNovix (left) and NanoDrop (right). The multiple R-squared (R^2^), Spearman correlation (R_S_), and corresponding p-values are provided. The line of equality is presented as a diagonal line. The dashed line corresponds to the linear regression model with formula as indicated. For each ratio, the proposed reference interval is indicated where DNA is regarded as pure (A_260/280_: [1.7,2.0], A_260/230_: [2.0,2.2]). T_1_, timepoint 1 (before freeze storing); T_2_, timepoint 2 (after freeze storing). | |
